# Antibiotic leftovers in primary health care: an approach to economic impact and pharmaceutical care^*^

**DOI:** 10.1590/1980-220X-REEUSP-2026-0263en

**Published:** 2026-08-07

**Authors:** Mariana Lino de Oliveira Santos, Maria Clara Padoveze

**Affiliations:** 1 Universidade de São Paulo, Escola de Enfermagem, São Paulo, SP, Brazil.

**Keywords:** Drug Utilization, Drug Resistance, Microbial, Primary Health Care

## Abstract

**Objective:**

To evaluate the results of antibiotic dispensing in a Primary Health Care Unit in the municipality of São Paulo and to explore users’ knowledge regarding the use and disposal of antibiotics through pharmaceutical consultation.

**Method:**

A prospective, quantitative study was conducted between January and April 2024, through the analysis of oral antibiotic prescriptions and pharmaceutical consultations using a semi-structured questionnaire.

**Results:**

It was identified that 81.9% (n = 1,225) of 1,495 prescriptions resulted in leftover medication, generating a waste of 8,061 pharmaceutical units and an estimated financial loss of R$2,349.54. Among the 17 users interviewed, there was low knowledge about antimicrobial resistance, medication use and disposal. Only 29.4% (n = 5) returned the leftover medication after pharmaceutical intervention.

**Conclusion:**

Dispensing medication with leftovers encourages the improper use and disposal of antibiotics, contributing to antimicrobial resistance. Pharmaceutical care, within a multidisciplinary team approach, has the potential to reduce waste and promote rational use. We propose the implementation of unit-dose packaging protocols and educational initiatives for professionals and users.

## INTRODUCTION

Antimicrobial resistance (AMR) represents a growing threat to global public health. Although the impact of AMR is widely studied in hospital settings, in primary health care (PHC) this impact is still insufficiently explored. As the primary level of healthcare, PHC faces significant challenges related to the inappropriate use of antibiotics. This irrational and excessive use contributes to AMR, increased expenses, and wasted resources^(1,2,3,4)^.

In PHC, antibiotic dispensing may include leftovers, which are excess quantities dispensed to the user that are not necessary for the prescribed treatment. These leftovers occur due to the lack of purchase of split-dose packaging and the technical difficulty of proper unit-dose dispensing in PHC. Dispensing medication with leftovers directly encourages inappropriate use of medications, such as self-medication or sharing with people with similar symptoms, and increases the possibility of environmental contamination through improper disposal, contributing to the spread of AMR in the community. Furthermore, it contributes to a significant waste of financial resources in the healthcare system^(5)^.

The Rational Use of Medicines (RUM), according to the World Health Organization (WHO), is when the user receives the appropriate medication for their condition, in correct and adjusted doses that meet their own individual requirements, ensuring the adequate duration of treatment at a reasonable cost for both the user and the community. The effective application of RUM represents a significant challenge in combating AMR^(6)^.

Adopting strategies within the multidisciplinary team that support RUM is fundamental. In PHC, the pharmacist works in an integrated manner with the multidisciplinary team, strengthening the bond with the community and playing an important role in combating AMR. Their responsibilities include pharmaceutical consultations, participation in educational groups, home visits, shared consultations, and Individual Therapeutic Projects (ITPs), especially for complex cases. Therefore, the pharmacist can significantly contribute to minimizing the factors that contribute to AMR by promoting guidance and educational actions that enhance RUM and proper disposal^(7)^. These actions are integrated in a multidisciplinary way as part of a collective effort to address AMR.

Finally, this study aims to evaluate the results of antibiotic dispensing in a Primary Health Care Unit (*UBS*) in the municipality of São Paulo, as well as to explore users’ knowledge regarding the use and disposal of antibiotics through pharmaceutical consultation.

## METHOD

### Design of Study

This is a prospective longitudinal study with a quantitative approach on the intervention of pharmaceutical care in the rational use of antibiotics.

### Study Local and Period

The study was carried out in *UBS Parque Araribá*, linked to the Technical Health Supervision of Campo Limpo, in the municipality of São Paulo – the largest city in the country, with an estimated population of 11,895,578 inhabitants. The *UBS* has 36,363 registered users (data from August 2024) and ten Family Health Strategy teams. Data collection took place between January 1st and April 30th, 2024.

### Population and Selection Criteria

The study population consisted of adult PHC users who received antibiotic prescriptions from consultations with health professionals (doctors, dentists, or nurses) at the unit. The study included individuals with prescriptions for solid oral antibiotics (tablets, capsules, and coated tablets) available at the service, according to the Municipal List of Medicines (REMUME), whose pharmaceutical presentations did not allow for precise dispensing for the prescribed treatment, leading to dispensing with leftovers^(8)^. Within this criterion, ten medications were identified: amoxicillin 500mg, cephalexin 500mg, ciprofloxacin 500mg, clarithromycin 500mg, clindamycin 300mg, doxycycline 100mg, metronidazole 250mg, nitrofurantoin 10mg, norfloxacin 400mg, and sulfamethoxazole 400mg + trimethoprim 80mg.

### Sample Definition

A convenience sample was defined sequentially, in the order of service at the *UBS*. The researcher dedicated one day a week over a 15-week period to consultations and dispensing in an office reserved for this purpose. This demand resulted in 17 consultations with 18 dispensings, representing 1.2% (n = 1,495) of the dispensings during the study period.

### Data Collection Procedure

In the first stage of the research, 1,495 prescriptions (second copies) of oral and solid antibiotics, available in the service and dispensed with leftovers, were analyzed for investigation of the antibiotic dispensing profile. In this stage, the volume of Pharmaceutical Units (PU) resulting from the leftovers was calculated by the difference between the quantity dispensed and the quantity prescribed for the treatment. The value of the financial losses was calculated based on the unit price shown on the invoice multiplied by the quantity of leftover medication, allowing for an estimate of the losses projected for subsequent four-month periods. The potential for waste was calculated using the total number of PUs dispensed compared to the total PUs wasted (leftovers) of each antibiotic presentation.

The second stage involved pharmaceutical consultations with service users who had been prescribed antibiotics. This took place simultaneously with the first stage, aiming at testing the feasibility of pharmaceutical care intervention in this format. In this phase, 17 users were assisted, who were questioned about their prior knowledge of antibiotics and given guidance based on their answers and doubts. The medications were dispensed according to the prescription, with specific instructions given to the user regarding the return of any leftover antibiotics. At this time, a follow-up appointment was scheduled for a second consultation where the quantities of leftover items returned were evaluated and the outcome of the intervention was assessed.

### Data Analysis and Treatment

Data were collected using a pharmacotherapeutic formulary as a support tool and subsequently entered into a spreadsheet using the software *Microsoft Excel.* The form was developed specifically for this research to guide the questions directed to the user. It included questions about prior knowledge of antibiotics, antimicrobial resistance, and medication disposal; information about the dispensed medication and any leftover medication; and questions related to the post-intervention consultation, specifically when the user returns with any leftover medication. The possible answers were yes, no, I don’t know. Finally, there was an open field for noting important statements related to the questions. The questionnaire is available in the supplementary file. Data were analyzed using descriptive statistics, and absolute and relative distribution of the variables studied.

### Ethical Aspects

The study was submitted to the ethics committee of the Nursing School at the Universidade de São Paulo (USP), opinion number 6,544,357, via Plataforma Brasil, in accordance with current legislation, with authorization for conducting the research CAAE number 74545323.4.0000.5392.

## RESULTS

The results obtained are presented according to the stages mentioned in the methods section.

### Stage 1 – Antibiotic Dispensing Profile

The result demonstrated that of the 1,495 prescriptions analyzed, 1,225 (81.9%) were dispensed with PUs in quantities exceeding those required for treatment. Among these prescriptions dispensed with leftovers, 51.4% (n = 630) came from the *UBS* itself, followed by 28.1% (n = 344) from private services and 20.5% (n = 251) from other public health services.

By observing all prescriptions for the ten antibiotics listed for the study and relating the amount wasted (leftover) to the amount dispensed, the waste potential of each medication was determined (Table 1). For example, it can be noted that the drug norfloxacin 400 mg had the greatest potential for waste, with an estimated 28.8% of all medication dispensed during the period (n = 204) being wasted.

**Table 1:** Total pharmaceutical units (PUs) dispensed and wasted, according to antibiotic type and waste potential based on prescriptions filled between January and April 2024 at the *UBS Parque Araribá* , N = 43,556 PUs dispensed – São Paulo, SP, Brazil, 2024.

Antibiotic	Dispensed PUs (n = 43,556)	Wasted PUs (n = 8,061)	Potential for losses (%)
Amoxicillin 500 mg	17972	4460	24.8%
Cephalexin 500 mg	11470	1212	10.6%
Ciprofloxacin 500 mg	2825	349	12.4%
Clarithromycin 500 mg	1470	132	9.0%
Clindamycin 300 mg	1568	63	4.0%
Doxycycline 100mg	1110	78	7.0%
Metronidazole 250 mg	4760	1284	27.0%
Nitrofurantoin 100 mg	372	80	21.5%
Norfloxacin 400 mg	709	204	28.8%
Sulfamethoxazole+trimetropine 800/160 mg	1300	199	15.3%

Source: Prepared by the author.

With a leftover volume of 4,460 PUs during the period, amoxicillin 500 mg was the medication that contributed most to the loss of financial resources, reaching an estimated R$ 936.60 for the four months of the study. Nitrofurantoin 100 mg, which was dispensed in excess in all prescriptions, contributed to the waste of 80 PUs out of the 372 total PUs dispensed of this medication, generating an estimated financial loss of R$ 25.60. Doxycycline 100 mg was the medication that resulted in the smallest financial loss, calculated at R$ 22.62. Adding up all the losses, the total amount obtained for the period was R$ 2,349.54 (Table 2).

**Table 2:** Estimate of Financial resources wasted (in R$) through leftover medications between January and April 2024 at the *UBS Parque Araribá* – São Paulo, SP, Brazil, 2024.

Antibiotic	Wasted PU (leftover)	Price per PU^*^	Estimated wasted value (R$)
Amoxicillin 500 mg	4,460	0.21	936.6
Cephalexin 500 mg	1,212	0.52	630.24
Ciprofloxacin 500 mg	349	0.17	59.33
Clarithromycin 500 mg	132	1.74	229.68
Clindamycin 300 mg	63	1.1	69.3
Doxycycline 100 mg	78	0.29	22.62
Metronidazole 250 mg	1,284	0.18	231.12
Nitrofurantoin 100 mg	80	0.32	25.6
Norfloxacin 400 mg	204	0.37	75.4
Sulfamethoxazole+trim.800/160 mg	199	0.35	69.65
**TOTAL**	**8,061**	**–**	**2,349.54**

Source: Prepared by the author.

*Average price – as described in invoices for the period.

### 2nd Stage – Antibiotic Dispensing Profile

**Figure 1:**
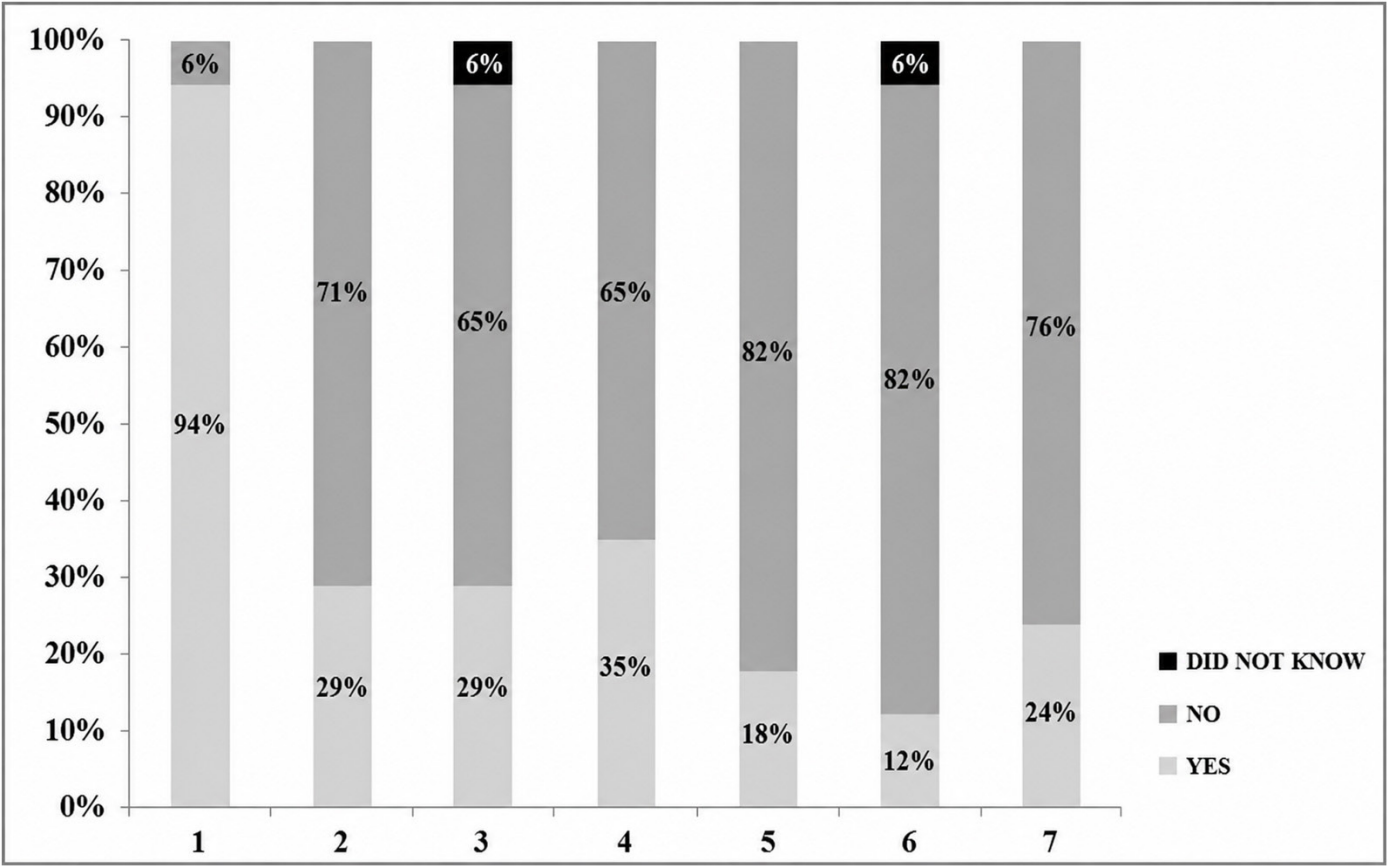
Percentage distribution of users’ responses regarding knowledge about antibiotics, collected during pharmaceutical consultations from January to April 2024 at the UBS *Parque Araribá*, São Paulo/SP. N = 17.

Source: Prepared by the author.

Legend: The x-axis, referring to the questions: 1. Do you know the reason for the prescription?; 2. Have you ever been unsure how to administer medication?; 3. Do you know what antimicrobial resistance is?; 4. Do you know what an antibiotic is?; 5. Have you used antibiotics without a prescription this year?; 6. Do you usually keep antibiotics at home?; 7. Do you know where to dispose of leftover or expired items?

Seventeen patients were treated, with 18 antibiotics dispensed, some with leftovers. The results obtained showed that only one user did not know the reason for the prescription (Figure 1). Among the users assisted in the pharmaceutical consultation, five have had at least some doubt about prescriptions at some point, five were able to briefly explain what AMR is, and six reported knowledge about antibiotics. Three people used antibiotics without a prescription in the last year, 14 reported not having antibiotics at home, and 13 people said they did not know where to dispose of leftover or expired medications.

The pharmacotherapeutic formulary used for consultations consisted of axes 1 and 2, correlated to the consultation times.

### Axis 1: Knowledge About Antibiotics

The moment of the first consultation, where the user’s prior knowledge is assessed with questions related to antibiotics, AMR, and disposal (Figure 1).

### Axis 2: Profile of Leftovers and Returns

These data relate to the second consultation (follow-up). Among the users who were seen in the first pharmaceutical consultation, only five users (29.4%) returned to give the leftover medication back, resulting in compliance after the pharmaceutical intervention regarding the correct return of the leftover medication. Of these five, only one individual reported not completing the treatment according to the prescription and instructions, even though the amount returned was in accordance with what was agreed upon initially.

## DISCUSSION

This study shows that there were leftovers in more than 80% of antibiotic prescriptions at the *UBS Parque Araribá* due to their presentation (packaging) during the study period. These leftovers are relatively large and can contribute to the spread of AMR in the community. However, in Brazil, there are no policies that effectively regulate this dispensing based on the RUM, where, according to the WHO, the user should receive the appropriate medication in the correct dose, adjusted to their requirements, guaranteeing complete treatment at a reasonable cost.

The leftovers, analyzed from an economic standpoint, indicated a financial loss of R$ 2,349.54 during the period, with a projection of more than R$ 7,000.00 in 2024 for the *UBS Parque Araribá* alone. These figures may not seem large, but when applied on a larger scale, can be estimated to represent significant losses. A similar study in the municipality of Guarulhos, in 2020, estimated a loss of approximately R$ 2,530.26 in an *UBS* that year, projecting an estimated waste of R$ 888,272.04 for the year in this municipality due to leftovers^(2)^. The municipality of São Paulo has approximately 500 health services that dispense medication with the possibility of leftovers (*UBS*s and AMAs). Comparing this to the study in Guarulhos, we can assume that São Paulo may experience an even more significant financial loss, and these resources could be allocated to purchasing more medications or to other investments.

Given the current scenario of scarcity, it is increasingly unacceptable that essential resources, such as antibiotics, are wasted thoughtlessly due to inaction by health authorities. Above all, controlling antibiotic use is imperative in the fight against AMR.

However, the harm resulting from antibiotics leftovers goes beyond the purely financial aspect, when one considers that these are in the hands of a relatively uninformed population. Our findings demonstrated that a significant portion of those interviewed did not know what an antibiotic or AMR was, nor did they know where to dispose of treatment leftover or expired medications. Even though they were instructed to return to give back the leftovers, this activity was carried out by only a small fraction of the individuals who received the instruction. As a consequence of the antibiotics leftovers in the possession of the population, one can anticipate the potential for misuse of these leftovers by the users themselves or by third parties (neighbors, relatives, children), considering that there is not widespread knowledge of the associated risks and there is low awareness about the proper use of antibiotics^(5)^. In this sense, the need for a multidisciplinary approach to patient care in primary care is highlighted, recognizing the role of professionals beyond the traditional prescriber, who is the medical professional. To achieve this, teams have to be prepared to carry out these educational actions, taking into account the profile and needs of the patients served^(9)^.

Another important factor that deserves being underscored is the improper disposal of antibiotics. This represents a significant risk to public health, contributes to AMR through contamination of the environment (soil, water, and animals), and can spread to an entire community. Poor sanitation conditions and social vulnerability, coupled with misinformation, contribute to environmental contamination by antibiotics^(5)^. A study in Minas Gerais detected the presence of these pharmaceuticals in all water samples, including in areas with little human interference, indicating that their spread extends beyond urban centers^(10)^. This highlights the importance of educating the population not only about the use, but also about their proper disposal^(11)^. Other authors also state that non-compliant antibiotic packaging generates a large amount of waste in the community, contributing to AMR as a health, social, and economic problem^(12,13)^.

In response to this situation of leftovers, unit-dose packaging and precise dispensing seem to be the most appropriate solution, standing out as a significant tool. Individualized dispensing allows the user to receive only the exact amount needed for treatment. An experiment in community pharmacies in France (2014-2015) replaced the dispensing of 14 antibiotics in their original packaging with unit-dose packaging, reducing the amount dispensed, minimizing waste, and improving adherence to treatment^(13,14)^. However, in Brazil, unit-dose dispensing of medications can only be done at the point of dispensing, under controlled conditions, according to RDC No. 80, of May 11, 2006, which requires investment and significant adaptation of services^(15)^. Another way to promote the unit-dose dispensing of antibiotics would be to present these medications in divisible blister packs; however, there are few brands and types of antibiotics on the market in the country that do this.

Nevertheless, in Brazil there are no measures to regulate the production of drug presentations in packaging that is compatible with the interests of public health, nor are there policies to regulate the adequacy of the pharmaceutical market and encourage health services to acquire antibiotics in unit doses or in fractionable packaging, which would guarantee individualized dispensing. This would be the ideal model for acquiring antibiotics in the Brazilian healthcare system, ensuring the exact dosage for the treatment prescribed.

Until this broader and more sensible solution is implemented, a possible alternative would be to establish sanitary conditions for unit-dose packaging on-site in each *UBS* pharmacy (or in a central or regional hub), even if it generates additional operational costs and work processes. However, such investments could be diluted by the sustainability of more accurate dispensing.

The pharmacist plays an important role as a clinical professional, intervening in improving adherence to and management of these medications^(12)^. Pharmaceutical interventions aim to ensure comprehensive therapeutic care for the user. However, they face challenging cultural obstacles, such as a lack of recognition of the importance of the correct use of medications and pharmaceutical care, an important part of their care and treatment^(16)^. Furthermore, it is essential that professionals are qualified and fully aware of the strategic importance of their roles. Accordingly, a UK author points to the need to prepare pharmaceutical professionals to deal with AMR through the conscious administration of antimicrobials, including professional undergraduate programs that incorporate subjects focused on antimicrobial administration as a mandatory competency in pharmacy schools in that country, aligning education with practice, which could be perfectly applicable to the Brazilian context^(17)^. We invariably emphasize that pharmaceutical consultations can complement the efforts of nurses and other healthcare professionals in providing guidance to users and their families, thus enhancing teamwork.

Therefore, awareness campaigns, education, and public policy incentives are necessary to preserve the effectiveness of antibiotics for future generations^(18)^.

As long as the practice of dispensing with leftovers continues in healthcare services, financial waste and the potential for increased AMR will persist. It is evident that pharmaceutical care, integrated with a multidisciplinary team, can reduce waste and promote RUM. Given this scenario, without the possibility of individualized dispensing, other measures must be taken in an attempt to minimize the irrational use and improper disposal of antibiotics. Therefore, the aim is to develop effective interventions to raise awareness among healthcare professionals and the general population about AMR and to mitigate its risk factors.

Considering the findings of this study, the development of technical support material aimed at standardizing the unitdose dispensing procedure and implementing a protocol for the individualized dispensing of antibiotics within the scope of PHC was proposed. This initiative aims to further enhance the pharmacist’s role in care, promoting greater adherence to treatment, minimizing the risks of environmentally inappropriate disposal, and reducing the possibility of misuse and waste of financial resources, through the dispensing of antibiotics in strict accordance with the prescribed dosage; consequently, to contribute to mitigating the impacts of leftover antibiotics on AMR in the community. This material is available on a webpage^(19)^.

## CONCLUSION

This study demonstrated the occurrence of leftover in antibiotics dispensing of healthcare facilities. The results of the pharmaceutical consultation demonstrated that there is insufficient knowledge about the use and disposal of antibiotics. Unit-dose dispensing of medications in PHC, combined with educational initiatives, can have great potential to reduce waste and contribute to mitigating AMR.

## Data Availability

All the data supporting the results of this study were published in the article itself.
