# Supplementary material for: Antibiotic leftovers in primary health care: an approach to economic impact and pharmaceutical care
Source: Rev Esc Enferm USP. 2026 Aug 7;60:e20260263. doi: 10.1590/1980-220X-REEUSP-2026-0263en (PMC13451054; doi:10.1590/1980-220X-REEUSP-2026-0263en)
Supplement: Translation

# Translation: Sobras de antibióticos na atenção primária à saúde: abordagem sobre impacto econômico e o cuidado farmacêutico^*^

**DOI:** 10.1590/1980-220X-REEUSP-2026-0263pt

**Authors:** Mariana Lino de Oliveira Santos, Maria Clara Padoveze

**Affiliations:** 1 Universidade de São Paulo, Escola de Enfermagem, São Paulo, SP, Brasil.

**Keywords:** Uso de Medicamentos, Resistência Microbiana a Medicamentos, Atenção Primária à Saúde

## Abstract

**Objetivo:**

Avaliar os resultados da dispensação de antibióticos em uma Unidade Básica de Saúde do município de São Paulo e explorar o conhecimento de usuários a respeito do uso e descarte de antibióticos por meio da consulta farmacêutica.

**Método:**

Estudo prospectivo, quantitativo, realizado entre janeiro e abril de 2024, por meio da análise de prescrições de antibióticos orais e consultas farmacêuticas em questionário semiestruturado.

**Resultados:**

Identificou-se que 81,9% (n = 1.225) de 1.495 prescrições resultaram em sobras, gerando desperdício de 8.061 unidades farmacêuticas e perda financeira estimada em R$2.349,54. Entre os 17 usuários entrevistados, verificou-se baixo conhecimento sobre resistência microbiana, uso e descarte de medicamentos. Apenas 29,4% (n = 5) devolveram as sobras após intervenção farmacêutica.

**Conclusão:**

A dispensação com sobras favorece o uso e descarte inadequados dos antibióticos, contribuindo para a resistência antimicrobiana. O cuidado farmacêutico, dentro de uma abordagem na equipe multiprofissional, tem potencial para reduzir desperdícios e promover o uso racional. Propõe-se a implantação de protocolos de unitarização e ações educativas para profissionais e usuários.

## INTRODUÇÃO

A Resistência Antimicrobiana (RAM) representa uma ameaça crescente à saúde pública global. Embora o impacto da RAM seja amplamente estudado em ambientes hospitalares, na atenção primária à saúde (APS) esse impacto é ainda insuficientemente explorado. Como primeiro nível de atenção em saúde, a APS enfrenta desafios significativos relacionados ao uso inadequado de antibióticos. Este uso não racional e excessivo contribui para a RAM, aumento dos gastos e desperdício de recursos^(1,2,3,4)^.

Na APS, a dispensação de antibióticos pode conter sobras, que são as quantidades excedentes dispensadas ao usuário e que não são necessárias para o tratamento prescrito. Estas sobras ocorrem devido à não aquisição de embalagens fracionáveis e à dificuldade técnica de fracionamento da forma adequada na APS. A dispensação com sobras favorece diretamente usos inadequados dos medicamentos, como a automedicação ou compartilhamento com pessoas com sintomas semelhantes e aumentam a possibilidade de contaminação do meio ambiente por meio do descarte impróprio, contribuindo para a disseminação da RAM na comunidade. Além disso, concorre para significativo desperdício de recursos financeiros do sistema de saúde^(5)^.

O Uso Racional de Medicamentos (URM) de acordo com a Organização Mundial da Saúde (OMS) é quando o usuário recebe o medicamento apropriado para sua condição, nas doses certas e ajustadas às suas necessidades individuais, garantindo a duração adequada do tratamento a um custo razoável tanto para o usuário quanto para a comunidade. A aplicação efetiva do URM representa um desafio significativo no combate à RAM^(6)^.

A adoção de estratégias, no contexto da equipe multiprofisisonal, que apoiem o URM é fundamental. Na APS, o farmacêutico atua de forma integrada à equipe multiprofissional, fortalecendo o vínculo com a comunidade e exercendo um papel importante no combate à RAM. Dentre suas atribuições estão a consulta farmacêutica, participação em grupos educativos, visitas domiciliares, consultas compartilhadas e em Projetos Terapêuticos Singulares (PTS), especialmente para os casos complexos. Desta forma, o farmacêutico pode contribuir significativamente para minimizar os fatores que corroboram a RAM promovendo orientações e ações educativas que potencializam o URM e o descarte correto^(7)^. Essas ações integram-se de maneira multidisciplinar no esforço coletivo para o enfrentamento da RAM.

Por fim, este estudo tem como objetivo avaliar os resultados da dispensação de antibióticos em uma Unidade Básica de Saúde (UBS) do município de São Paulo, bem como explorar o conhecimento de usuários a respeito do uso e descarte de antibióticos por meio da consulta farmacêutica.

## MÉTODO

### Desenho do Estudo

Trata-se de um estudo longitudinal prospectivo de abordagem quantitativa sobre a intervenção do cuidado farmacêutico no uso racional de antibióticos.

### Local e Período do Estudo

Realizado na UBS Parque Araribá, vinculada à Supervisão Técnica de Saúde do Campo Limpo, no município de São Paulo – a maior cidade do país, com população estimada em 11.895.578 habitantes. A UBS possui 36.363 usuários cadastrados (dados de agosto/2024) e dispõe de dez equipes da Estratégia Saúde da Família. A coleta de dados ocorreu no período entre 1º de janeiro e 30 de abril de 2024.

### População e Critérios de Seleção

A população do estudo foi composta por usuários adultos da APS que receberam prescrições de antibióticos provenientes das consultas dos profissionais de saúde (médicos, dentistas ou enfermeiros) da unidade. Foram incluídos na pesquisa os indivíduos com prescrição de antibióticos para uso oral sólido (comprimidos, cápsulas e drágeas) disponíveis no serviço, considerando a Relação Municipal de Medicamentos (REMUME), cujas apresentações farmacêuticas não permitiam a dispensação exata para o tratamento prescrito, levando a dispensação com sobras^(8)^. Dentro deste critério, identificaram-se dez medicamentos: amoxicilina 500mg, cefalexina 500 mg, ciprofloxacino 500mg, claritromicina 500mg, clindamicina 300mg, doxiciclina 100mg, metronidazol 250mg, nitrofurantoína 10 mg, norfloxacino 400 mg e sulfametoxazol 400mg + trimemiddlerima 80mg.

### Definição da Amostra

Definiu-se uma amostra por conveniência e sequencial, na ordem de atendimento na UBS. A pesquisadora dedicou um dia da semana no período de 15 semanas para consultas e dispensações em consultório reservado para este fim. Esta demanda resultou em 17 atendimentos com 18 dispensações, o que representou 1,2% (n = 1.495) das dispensações do período do estudo.

### Procedimento de Coleta de Dados

Na primeira etapa da pesquisa foram analisadas 1.495 prescrições (segundas vias) dos antibióticos de uso oral e sólidos, disponíveis no serviço e dispensados com sobras, com o objetivo de investigar o perfil de dispensação dos antibióticos. Nessa etapa, o volume de Unidades Farmacêuticas (UF) provenientes das sobras foi calculado mediante a diferença entre a quantidade dispensada e a quantidade prescrita para o tratamento. O valor das perdas financeiras foi calculado com base no preço unitário presente em nota fiscal multiplicado pela quantidade de sobra de cada medicamento, sendo possível uma estimativa das perdas com projeção para os quadrimestres subsequentes. O potencial de desperdício foi calculado utilizando os valores totais de UFs dispensadas em comparação às UFs totais desperdiçadas (sobras) de cada uma das apresentações dos antibióticos.

A segunda etapa foi a consulta farmacêutica aos usuários do serviço com prescrição de antibióticos, que aconteceu simultaneamente à primeira, com o objetivo de testar a viabilidade da intervenção do cuidado farmacêutico nesse formato. Nesta etapa, foram atendidos 17 usuários, os quais foram questionados sobre o conhecimento prévio de antibióticos e orientados conforme suas respostas e dúvidas. Foram dispensados os medicamentos de acordo com a prescrição, orientando especificamente o usuário sobre a devolução das sobras dos antibióticos. Ainda neste momento, foi agendada data de retorno para segunda consulta onde foram avaliadas as quantidades de sobras devolvidas e realizado o desfecho da intervenção.

### Análise e Tratamento dos Dados

Os dados foram coletados utilizando-se como instrumento para apoio o formulário farmacoterapêutico e, posteriormente, inseridos em planilha utilizando como ferramenta o software *Microsoft Excel.* O formulário foi desenvolvido especificamente para essa pesquisa de modo a nortear as perguntas direcionadas ao usuário. Nele havia perguntas sobre o conhecimento prévio sobre antibióticos, resistência antimicrobiana e descartes de medicamentos; informações sobre o medicamento dispensado e sobras; e perguntas relacionadas à consulta após intervenção, momento do retorno do usuário com as sobras dos medicamentos. As alternativas de respostas eram sim, não, não sei. Ao final, havia um campo aberto para anotação de falas importantes relacionadas às questões. O questionário está disponível no arquivo suplementar. Os dados foram analisados por meio de estatística descritiva, distribuição absoluta e relativa das variáveis estudadas.

### Aspectos Éticos

O estudo foi submetido ao comitê de ética da Escola de Enfermagem da Universidade de São Paulo (USP), parecer nº 6.544.357, via Plataforma Brasil, atentando às legislações vigentes, com a autorização para a realização da pesquisa CAAE nº 74545323.4.0000.5392.

## RESULTADOS

Os resultados obtidos são apresentados segundo as etapas citadas na seção de métodos.

### 1ª Etapa – Perfil de Dispensação dos Antibióticos

O resultado demonstrou que das 1.495 prescrições analisadas, 1.225 (81,9%) foram dispensadas com UFs em quantidades que excedem às necessárias para o tratamento. Entre estas prescrições dispensadas com sobras, 51,4% (n = 630) foram provenientes da própria UBS, seguidas de 28,1% (n = 344) de serviços particulares e 20,5% (n = 251) de outros serviços públicos de saúde.

Observando-se todas as prescrições dos dez antibióticos elencados para o estudo e relacionando a quantidade desperdiçada (sobra) com a quantidade dispensada, traçou-se o potencial de desperdício de cada medicamento (Tabela 1). Exemplificando, pode-se notar que o medicamento norfloxacino 400 mg teve o maior potencial de desperdício quando se estimou que 28,8% de todo medicamento dispensado no período (n = 204) foi desperdiçado.

**Tabela 1:** Unidades farmacêuticas (UFs) totais dispensadas e desperdiçadas, segundo o tipo de antibiótico e potencial de desperdício conforme prescrições atendidas entre janeiro e abril de 2024 na UBS Parque Araribá, N = 43.556 UFs dispensadas – São Paulo, SP, Brasil, 2024.

Antibiótico	UFs dispensadas (n = 43.556)	UFs desperdiçadas (n = 8.061)	Potencial de perdas (%)
Amoxicilina 500 mg	17972	4460	24,8%
Cefalexina 500 mg	11470	1212	10,6%
Ciprofloxacino 500 mg	2825	349	12,4%
Claritromicina 500 mg	1470	132	9,0%
Clindamicina 300 mg	1568	63	4,0%
Doxiciclina 100mg	1110	78	7,0%
Metronidazol 250 mg	4760	1284	27,0%
Nitrofurantoína 100 mg	372	80	21,5%
Norfloxacino 400 mg	709	204	28,8%
Sulfametoxazol+trimetropina 800/160 mg	1300	199	15,3%

Fonte: Elaborado pela Autora.

Com um volume de sobras em 4.460 UFs no período, a amoxicilina 500 mg foi o medicamento que mais colaborou com a perda de recursos financeiros, chegando a R$ 936,60, estimados para os quatro meses do estudo. A nitrofurantoína 100 mg, que teve dispensação com sobra em todas as prescrições, contribuiu para o desperdício de 80 UFs das 372 UFs totais dispensadas deste medicamento, gerando uma perda financeira estimada de R$ 25,60. A doxiciclina 100 mg foi o medicamento que obteve menor perda financeira, calculada em R$ 22,62. Somadas todas as perdas, obteve-se o total de R$ 2.349,54 para o período (Tabela 2).

**Tabela 2:** Estimativa de recurso financeiro desperdiçado (em R$) por meio das sobras dos medicamentos entre janeiro e abril de 2024 na UBS Parque Araribá – São Paulo, SP, Brasil, 2024.

Antibiótico	Uf desperdiçadas (sobras)	Preço por Uf^*^	Valor desperdiçado estimado (R$)
Amoxicilina 500 mg	4.460	0,21	936,6
Cefalexina 500 mg	1.212	0,52	630,24
Ciprofloxacino 500 mg	349	0,17	59,33
Claritromicina 500 mg	132	1,74	229,68
Clindamicina 300 mg	63	1,1	69,3
Doxiciclina 100 mg	78	0,29	22,62
Metronidazol 250 mg	1.284	0,18	231,12
Nitrofurantoína 100 mg	80	0,32	25,6
Norfloxacino 400 mg	204	0,37	75,4
Sulfametoxazol+trim.800/160 mg	199	0,35	69,65
**TOTAL**	**8.061**	**–**	**2.349,54**

Fonte: Elaborado pela Autora.

*Preço médio - conforme descrito em notas fiscais do período

### 2ª Etapa – Perfil de Dispensação dos Antibióticos

Foram atendidos 17 usuários, com dispensação de 18 antibióticos com sobras. Os resultados obtidos demonstraram que apenas um usuário não sabia o motivo da prescrição (Figura 1). Entre os usuários atendidos na consulta farmacêutica, cinco já tiveram alguma dúvida com prescrição em algum momento, cinco souberam dizer de maneira resumida o que é RAM e seis informaram conhecimento sobre antibiótico. Três utilizaram antibióticos sem receita no último ano, 14 relataram não ter antibióticos em casa e 13 pessoas disseram não saber onde descartar medicamentos que sobram de tratamentos ou vencidos.

**Figura 1:** Distribuição percentual de respostas dos usuários, referentes ao conhecimento sobre antibióticos, coletadas em consulta farmacêutica no período de janeiro a abril de 2024 na UBS Parque Araribá, São Paulo/SP. N = 17.

Fonte: Elaborado pela Autora.

Legenda: Eixo x, referente as perguntas: 1. Sabe motivo da prescrição?; 2. Já teve dúvida de como administrar medicamento?; 3. Sabe o que é resistência antimicrobiana?; 4. Sabe o que é antibiótico?; 5. Usou antibiótico sem receita este ano?; 6. Costuma ter antibióticos em casa?; 7. Sabe onde descartar sobras ou vencidos?

O formulário farmacoterapêutico usado para as consultas era composto de eixos 1 e 2, correlacionados aos momentos das consultas.

### Eixo 1: Conhecimento Sobre Antibióticos

Momento da primeira consulta, onde é avaliado o conhecimento prévio do usuário com perguntas relacionadas aos antibióticos, RAM e descarte (Figura 1).

### Eixo 2: Perfil das Sobras e Devolução

São os dados relacionados à segunda consulta (retorno). Dentre os usuários que foram atendidos na primeira consulta farmacêutica, apenas cinco usuários (29,4%) retornaram para a devolução das sobras, resultando em conformidade após a intervenção farmacêutica referente à devolução correta das sobras. Dentre estes cinco, apenas um indivíduo reportou não concluir o tratamento de acordo com a prescrição e orientação, ainda que a quantidade devolvida estivesse em acordo com a pactuada no primeiro momento.

## DISCUSSÃO

Este estudo mostra que houve sobra em mais de 80% das prescrições de antibióticos na UBS Parque Araribá devido a sua apresentação (embalagem) no período estudado. Estas sobras são relativamente grandes, e podem contribuir para disseminação da RAM na comunidade. Contudo, não há no Brasil políticas que regulamentem de maneira efetiva esta dispensação com base no URM, onde, segundo a OMS, o usuário deve receber o medicamento apropriado em dose correta, ajustado às suas necessidades, garantindo o tratamento completo a um custo razoável.

As sobras analisadas do ponto de vista econômico indicaram uma perda financeira de R$ 2.349,54 no período, com projeção de mais de R$ 7.000,00 em 2024 somente na UBS Parque Araribá. Estes valores não se destacam por ser um montante vultoso, mas quando aplicados em escala podem-se estimar perdas significativas. Um estudo similar no município de Guarulhos, em 2020, obteve estimativa de perda de aproximadamente R$ 2.530,26 em uma UBS naquele ano, o que projetou para este município um desperdício com as sobras estimado de R$ 888.272,04 para o ano^(2)^. O município de São Paulo é composto por cerca de 500 serviços de saúde que realizam dispensação com possibilidade de sobras (UBSs e AMAs). Comparando com o estudo de Guarulhos, podemos supor que São Paulo pode chegar a um valor de perda financeira ainda mais relevante, sendo que estes recursos poderiam ser destinados à compra de mais medicamentos ou a outros investimentos.

Diante de um cenário de escassez é cada vez menos aceitável que recursos essenciais, tais como os antibióticos, sejam desperdiçados de maneira irrefletida por inação das autoridades sanitárias. Sobretudo, o controle do consumo de antibióticos é um imperativo para o combate a RAM.

Contudo, o prejuízo consequente às sobras de antibióticos ultrapassa o aspecto meramente financeiro, quando se entende que estas estão em poder de uma população relativamente desinformada. Os nossos achados demonstraram que uma parcela importante dos entrevistados não soube responder o que era antibiótico ou RAM e também não sabiam onde descartar medicamentos provenientes de sobras de tratamentos ou vencidos. Mesmo tendo sido orientados a retornarem para devolução das sobras, essa atividade foi executada apenas por uma fração pequena dos indivíduos que receberam a orientação. Como consequência das sobras em posse da população, pode-se antecipar o potencial uso indevido destas sobras pelo próprio usuário ou terceiro (vizinho, parente, crianças), considerando que não há um amplo conhecimento dos riscos associados e há baixa conscientização sobre o uso adequado de antibióticos^(5)^. Nesse sentido, destaca-se a necessidade de um esforço multidisciplinar para abordagem dos pacientes na atenção primária, reconhecendo o papel de outros profissionais além do prescritor tradicional, que é o profissional médico. Para tal, as equipes precisam ser preparadas para exercer essas ações educativas, considerando o perfil e necessidades dos pacientes atendidos^(9)^.

Outro fator importante que merece destaque é o descarte inadequado de antibióticos. Este representa grande risco à saúde pública, contribui para a RAM por meio da contaminação do meio ambiente (solo, água, e animais) e pode estender-se a toda uma comunidade. Condições precárias de saneamento básico e vulnerabilidade social, aliadas à desinformação, colaboram para a contaminação ambiental por antibióticos^(5)^. Um estudo em Minas Gerais detectou a presença desses fármacos em todas as amostras de água, inclusive em áreas com pouca interferência humana, indicando que sua disseminação vai além dos centros urbanos^(10)^. Destaca-se, desta forma, a importância da educação da população não somente quanto ao uso, mas também quanto ao descarte adequado^(11)^. Outros autores também afirmam que a não conformidade das embalagens de antibióticos gera grande quantidade de resíduos na comunidade, contribuindo para a RAM como problema de saúde, social e econômico^(12,13)^.

Em reflexo a essa situação das sobras, a unitarização e dispensação exata parece ser a solução mais apropriada, destacando-se como uma ferramenta significativa. A dispensação individualizada possibilita que o usuário receba apenas a quantidade exata necessária para o tratamento. Um experimento em farmácias comunitárias da França (2014-2015) substituiu a dispensação de 14 antibióticos em embalagens originais pela unitarização, reduzindo a quantidade dispensada, minimizando desperdícios e favorecendo a adesão ao tratamento^(13,14)^. Contudo, a unitarização de medicamentos no Brasil só pode ser feita no local da dispensação, em condições controladas, de acordo com a RDC Nº 80, de 11 de maio de 2006, o que exige investimentos e relevante adaptação dos serviços^(15)^. Outra maneira de favorecer a dispensação unitarizada de antibióticos seria a apresentação destes medicamentos em cartelas fracionáveis, porém no país existem poucas marcas e tipos de antibióticos no mercado.

No entanto, no Brasil não há medidas para regulamentar a produção de apresentações medicamentosas em embalagens que sejam compatíveis com os interesses da saúde coletiva, bem como políticas que regulamentem a adequação do mercado farmacêutico e estimulem os serviços de saúde à aquisição de antibióticos unitarizados ou em embalagens fracionáveis, o que garantiria a dispensação individualizada. Esse seria o modelo ideal de aquisição de antibióticos no sistema de saúde brasileiro, assegurando a dispensação exata para o tratamento indicado pelo prescritor.

Enquanto essa solução mais ampla e sensata não acontece, uma alternativa possível seria estabelecer condições sanitárias para unitarização *in loco* em cada farmácia de UBS (ou em um pólo central ou regionalizado), ainda que gerando custos operacionais e processos de trabalho adicionais. Entretanto, tais investimentos poderiam se diluir pela sustentabilidade da dispensação mais acurada.

O farmacêutico tem importante papel como profissional clínico intervindo na melhoria da adesão e gerenciamento destes medicamentos^(12)^. As intervenções farmacêuticas visam garantir a assistência terapêutica integral ao usuário. Porém, deparam-se com obstáculos culturais desafiadores, como a falta de reconhecimento da importância do uso correto dos medicamentos e do atendimento farmacêutico, parte importante para o seu cuidado e tratamento^(16)^. Ainda, é fundamental que os profissionais sejam qualificados e tenham plena consciência da importância estratégica de suas funções. Em conformidade, um autor do Reino Unido aponta a necessidade de se prepararem profissionais farmacêuticos para lidar com a RAM por meio da administração consciente dos antimicrobianos, incluindo programas de graduação profissional que contenham na grade curricular disciplinas voltadas a administração de antimicrobianos como uma competência obrigatória em farmácias-escola daquele país, alinhando a educação com a prática, o que poderia ser perfeitamente aplicável ao contexto brasileiro^(17)^. Invariavelmente, destacamos que a consulta farmacêutica pode somar esforços à consulta do enfermeiro, e demais profissionais da saúde, no

quesito de orientação aos usuários e familiares, potencializando o trabalho de equipe.

Assim, fazem-se necessárias medidas de conscientização, educação e incentivo às políticas públicas para que seja preservada a eficácia dos antibióticos para gerações^(18)^.

Enquanto a dispensação com sobras continuar nos serviços de saúde, o desperdício financeiro e o potencial aumento da RAM persistirão. É evidente que o cuidado farmacêutico, integrado a equipe multidisciplinar, pode reduzir desperdícios e promover o URM. Diante desse cenário, sem a possibilidade de dispensação individualizada, outras medidas devem ser tomadas na tentativa de minimizar o uso irracional e descarte indevido dos antibióticos. Desta forma, propõe-se desenvolver intervenções eficazes para sensibilizar os profissionais da saúde e a população sobre a RAM e mitigar seus fatores de risco.

Considerando os achados deste estudo, propôs-se o desenvolvimento de material técnico de apoio voltado à padronização do procedimento de unitarização e à implantação de protocolo para a dispensação individualizada de antibióticos no âmbito da APS. Tal iniciativa objetiva qualificar ainda mais o papel do farmacêutico no cuidado, promovendo maior adesão ao tratamento,

minimizando riscos de descarte ambientalmente inadequado e reduzindo a possibilidade de uso indevido e desperdícios de recursos financeiros, por meio da dispensação dos antibióticos em conformidade estrita com a posologia prescrita. E, consequentemente, contribuir para a mitigação dos impactos das sobras de antibióticos sobre a RAM na comunidade. Este material está disponível em página eletrônica^(19)^.

## CONCLUSÃO

Este estudo demonstrou a ocorrência de sobras nas dispensações de antibióticos das unidades de saúde. Os resultados da consulta farmacêutica demonstraram que há conhecimento insuficiente sobre uso e descarte de antibióticos. A unitarização de medicamentos na APS, associada com ações educativas, pode ser de grande potencial para reduzir o desperdício e contribuir para a mitigação da RAM.

## Data Availability

Todo o conjunto de dados que dá suporte aos resultados deste estudo foi publicado no próprio artigo.

# Translation: Exceso de antibióticos en la atención primaria de salud: un enfoque sobre el impacto económico y la atención farmacêutica^*^

**DOI:** 10.1590/1980-220X-REEUSP-2026-0263es

**Authors:** Mariana Lino de Oliveira Santos, Maria Clara Padoveze

**Affiliations:** 1 Universidade de São Paulo, Escola de Enfermagem, São Paulo, SP, Brasil.

**Keywords:** Uso de Medicamentos, Resistência Microbiana a Medicamentos, Atenção Primária à Saúde

## Abstract

**Objetivo:**

Evaluar los resultados de la dispensación de antibióticos en una Unidad de Atención Primaria de Salud en el municipio de São Paulo y explorar el conocimiento de los usuarios sobre el uso y el desecho de antibióticos a través de la consulta farmacéutica.

**Método:**

Entre enero y abril de 2024 se realizó un estudio prospectivo y cuantitativo mediante el análisis de las prescripciones de antibióticos orales y las consultas farmacéuticas, utilizando un cuestionario semiestructurado.

**Resultados:**

Se identificó que el 81,9% (n = 1.225) de 1.495 recetas dieron como resultado medicamentos sobrantes, generando un desperdicio de 8.061 unidades farmacéuticas y una pérdida financiera estimada de R$2.349,54. Entre los 17 usuarios entrevistados, se observó un bajo nivel de conocimiento sobre la resistencia a los antimicrobianos, el uso de medicamentos y su correcto desecho. Solo el 29,4% (n = 5) devolvió la medicación sobrante tras la intervención farmacéutica.

**Conclusión:**

La dispensación de medicamentos con sobrantes fomenta el uso y el desecho inadecuados de antibióticos, lo que contribuye a la resistencia a los antimicrobianos. La atención farmacéutica, en el marco de un enfoque de equipo multidisciplinario, tiene el potencial de reducir el desperdicio y promover un uso racional. La propuesta incluye la implementación de protocolos de envasado en dosis unitarias e iniciativas educativas para profesionales y usuarios.

## INTRODUCCIÓN

La Resistencia a los Antimicrobianos (RAM) representa una amenaza creciente para la salud pública mundial. Aunque el impacto de la RAM se ha estudiado ampliamente en entornos hospitalarios, en la atención primaria de salud (APS) este impacto aún no se ha explorado lo suficiente. Como primer nivel de atención de salud, la APS enfrenta desafíos significativos relacionados con el uso inadecuado de antibióticos. Este uso irracional y excesivo contribuye a la RAM, al aumento de los gastos y al desperdicio de recursos^(1,2,3,4)^.

En la APS, la dispensación de antibióticos puede generar sobras, que son las cantidades excedentes entregadas al usuario y que no son necesarias para el tratamiento recetado. Estas sobras se producen debido a que no se adquieren envases fraccionables y a la dificultad técnica para fraccionar la dosis de manera adecuada en la APS.

La dispensación con sobras favorece directamente los usos inadecuados de los medicamentos, como la automedicación o el intercambio con personas con síntomas similares, y aumenta la posibilidad de contaminación del medio ambiente a través de la eliminación inadecuada, lo que contribuye a la diseminación de la RAM en la comunidad. Además, contribuye a un desperdicio significativo de los recursos financieros del sistema de salud^(5)^.

El Uso Racional de Medicamentos (URM), según la Organización Mundial de la Salud (OMS), se da cuando el usuario recibe el medicamento adecuado para su afección, en las dosis correctas y ajustadas a sus necesidades individuales, garantizando la duración adecuada del tratamiento a un costo razonable tanto para el usuario como para la comunidad. La aplicación efectiva del URM representa un desafío significativo en la lucha contra la RAM^(6)^.

Es fundamental adoptar estrategias, en el contexto del equipo multiprofesional, que respalden el URM. En la APS, el farmacéutico actúa de manera integrada en el equipo multiprofesional, fortaleciendo el vínculo con la comunidad y desempeñando un papel importante en la lucha contra la RAM. Entre sus atribuciones se encuentran la consulta farmacéutica, la participación en grupos educativos, las visitas a domicilio, las consultas compartidas y los Proyectos Terapéuticos Singulares (PTS), especialmente para los casos complejos. De esta manera, el farmacéutico puede contribuir significativamente a minimizar los factores que favorecen la RAM, promoviendo orientaciones y acciones educativas que potencien el URM y el desecho correcto^(7)^. Estas acciones se integran de manera multidisciplinaria en el esfuerzo colectivo para hacer frente a la RAM.

Por último, este estudio tiene como objetivo evaluar los resultados de la dispensación de antibióticos en una Unidad Básica de Salud (UBS) del municipio de São Paulo, así como explorar el conocimiento de los usuarios sobre el uso y la eliminación de antibióticos a través de la consulta farmacéutica.

## MÉTODO

### Diseño del Estudio

Se trata de un estudio longitudinal prospectivo de enfoque cuantitativo sobre la intervención de la atención farmacéutica en el uso racional de los antibióticos.

### Lugar y Período del Estudio

Realizado en la UBS Parque Araribá, adscrita a la Supervisión Técnica de Salud de Campo Limpo, en el municipio de São Paulo —la ciudad más grande del país, con una población estimada de 11 895 578 habitantes—. La UBS cuenta con 36 363 usuarios registrados (datos de agosto de 2024) y dispone de diez equipos de la Estrategia de Salud Familiar. La recolección de datos se llevó a cabo entre el 1 de enero y el 30 de abril de 2024.

### Población y Criterios de Selección

La población del estudio estuvo compuesta por usuarios adultos de la APS que recibieron recetas de antibióticos durante las consultas con los profesionales de la salud (médicos, dentistas o enfermeros) de la unidad. Se incluyeron en la investigación las personas con recetas de antibióticos para uso oral en forma sólida (tabletas, cápsulas y grageas) disponibles en el servicio, de acuerdo con la Lista Municipal de Medicamentos (REMUME), cuyas presentaciones farmacéuticas no permitían la dispensación exacta para el tratamiento recetado, lo que daba lugar a una dispensación con sobras^(8)^. Dentro de este criterio, se identificaron diez medicamentos: amoxicilina 500 mg, cefalexina 500 mg, ciprofloxacino 500 mg, claritromicina 500 mg, clindamicina 300 mg, doxiciclina 100 mg, metronidazol 250 mg, nitrofurantoína 10 mg, norfloxacino 400 mg y sulfametoxazol 400 mg + trimetoprima 80 mg.

### Definición de la Muestra

Se definió una muestra por conveniencia y secuencial, según el orden de atención en la UBS. La investigadora dedicó un día a la semana durante un período de 15 semanas a las consultas y dispensaciones en un consultorio reservado para este fin. Esta actividad dio lugar a 17 consultas con 18 dispensaciones, lo que representó el 1,2 % (n = 1 495) de las dispensaciones del período de estudio.

### Procedimiento de Recolección de Datos

En la primera etapa de la investigación se analizaron 1.495 recetas (duplicados) de antibióticos de uso oral y sólidos, disponibles en el servicio y dispensados con sobras, con el objetivo de investigar el perfil de dispensación de los antibióticos. En esta etapa, se calculó el volumen de Unidades Farmacéuticas (UF) provenientes de los excedentes mediante la diferencia entre la cantidad dispensada y la cantidad recetada para el tratamiento. El valor de las pérdidas financieras se calculó con base en el precio unitario que figura en la factura, multiplicado por la cantidad excedente de cada medicamento, lo que permitió realizar una estimación de las pérdidas con una proyección para los cuatrimestres siguientes. El potencial de desperdicio se calculó utilizando los valores totales de UF dispensadas en comparación con las UF totales desperdiciadas (sobrantes) de cada una de las presentaciones de los antibióticos.

La segunda etapa consistió en la consulta farmacéutica a los usuarios del servicio con receta de antibióticos, la cual se llevó a cabo simultáneamente a la primera, con el objetivo de evaluar la viabilidad de la intervención de atención farmacéutica en este formato. En esta etapa, se atendió a 17 usuarios, a quienes se les preguntó sobre sus conocimientos previos acerca

de los antibióticos y se les brindó orientación de acuerdo con sus respuestas y dudas. Se dispensaron los medicamentos de acuerdo con la receta, orientando específicamente al usuario sobre la devolución de los restos de antibióticos. Además, en ese momento se programó una fecha de regreso para una segunda consulta, en la que se evaluaron las cantidades de sobras devueltas y se realizó la evaluación final de la intervención.

### Análisis y Procesamiento de los Datos

Los datos se recopilaron utilizando como instrumento de apoyo el formulario farmacoterapéutico y, posteriormente, se ingresaron en una hoja de cálculo utilizando como herramienta el software *Microsoft Excel.* El formulario se desarrolló específicamente para esta investigación con el fin de orientar las preguntas dirigidas al usuario. En él se incluían preguntas sobre los conocimientos previos acerca de los antibióticos, la resistencia a los antimicrobianos y la eliminación de medicamentos; información sobre el medicamento dispensado y los restos; y preguntas relacionadas con la consulta posterior a la intervención, momento en el que el usuario regresaba con los restos de los medicamentos. Las opciones de respuesta eran «sí», «no» y «no sé». Al final, había un campo abierto para anotar comentarios importantes relacionados con las preguntas. El cuestionario está disponible en el archivo complementario. Los datos se analizaron mediante estadística descriptiva, distribución absoluta y relativa de las variables estudiadas.

### Aspectos Éticos

El estudio se presentó ante el comité de ética de la Escuela de Enfermería de la Universidad de São Paulo (USP), dictamen n.º 6.544.357, a través de la Plataforma Brasil, de conformidad con la legislación vigente, con la autorización para la realización de la investigación CAAE n.º 74545323.4.0000.5392.

## RESULTADOS

Los resultados obtenidos se presentan de acuerdo con las etapas mencionadas en la sección de métodos.

### 1.ª Etapa – Perfil de Dispensación de los Antibióticos

El resultado demostró que, de las 1.495 recetas analizadas, 1.225 (81,9 %) se dispensaron con unidades de dosis (UF) en cantidades que excedían las necesarias para el tratamiento. Entre estas recetas dispensadas con excedentes, el 51,4 % (n = 630) procedían de la propia Unidad Básica de Salud (UBS), seguidas por el 28,1 % (n = 344) de servicios privados y el 20,5 % (n = 251) de otros servicios públicos de salud.

Al analizar todas las recetas de los diez antibióticos incluidos en el estudio y relacionar la cantidad desperdiciada (sobrante) con la cantidad dispensada, se determinó el potencial de desperdicio de cada medicamento (Tabla 1). A modo de ejemplo, se puede observar que el medicamento norfloxacino 400 mg tuvo el mayor potencial de desperdicio, ya que se estimó que el 28,8 % de todo el medicamento dispensado en el período (n = 204) se desperdició.

**Tabla 1:** Unidades farmacéuticas (UF) totales dispensadas y desperdiciadas, según el tipo de antibiótico y el potencial de desperdicio, de acuerdo con las recetas atendidas entre enero y abril de 2024 en la UBS Parque Araribá, N = 43 556 UF dispensadas – São Paulo, SP, Brasil, 2024.

Antibiótico	UFs dispensadas (n = 43.556)	UFs desperdiciadas (n = 8.061)	Potencial de pérdidas (%)
Amoxicilina 500 mg	17972	4460	24,8%
Cefalexina 500 mg	11470	1212	10,6%
Ciprofloxacina 500 mg	2825	349	12,4%
Claritromicina 500 mg	1470	132	9,0%
Clindamicina 300 mg	1568	63	4,0%
Doxiciclina 100mg	1110	78	7,0%
Metronidazol 250 mg	4760	1284	27,0%
Nitrofurantoína 100 mg	372	80	21,5%
Norfloxacina 400 mg	709	204	28,8%
Sulfametoxazol+trimetropina 800/160 mg	1300	199	15,3%

Fuente: Elaborado por la Autora.

Con un volumen de sobras de 4.460 UFs en el período, la amoxicilina de 500 mg fue el medicamento que más contribuyó a la pérdida de recursos financieros, alcanzando los R$ 936,60, según las estimaciones para los cuatro meses del estudio. La nitrofurantoína de 100 mg, que se dispensó con excedentes en todas las recetas, contribuyó al desperdicio de 80 UF de las 372 UF totales dispensadas de este medicamento, lo que generó una pérdida financiera estimada de R$ 25,60. La doxiciclina de 100 mg fue el medicamento que registró la menor pérdida financiera, calculada en R$ 22,62. Al sumar todas las pérdidas, se obtuvo un total de R$ 2.349,54 para el período (Tabla 2).

**Tabla 2:** Estimación de los recursos financieros desperdiciados (en R$) debido a los excedentes de medicamentos entre enero y abril de 2024 en la UBS Parque Araribá – São Paulo, SP, Brasil, 2024.

Antibiótico	Uf desperdiciadas (sobras)	Precio por Uf^*^	Valor desperdiciado estimado (R$)
Amoxicilina 500 mg	4.460	0,21	936,6
Cefalexina 500 mg	1.212	0,52	630,24
Ciprofloxacina 500 mg	349	0,17	59,33
Claritromicina 500 mg	132	1,74	229,68
Clindamicina 300 mg	63	1,1	69,3
Doxiciclina 100 mg	78	0,29	22,62
Metronidazol 250 mg	1.284	0,18	231,12
Nitrofurantoína 100 mg	80	0,32	25,6
Norfloxacina 400 mg	204	0,37	75,4
Sulfametoxazol+trim.800/160 mg	199	0,35	69,65
**TOTAL**	**8.061**	**–**	**2.349,54**

Fuente: Elaborado por la Autora.

*Precio promedio: según lo indicado en las facturas del período

### 2.ª Etapa: Perfil de Dispensación de Antibióticos

Se atendió a 17 usuarios, a quienes se les dispensaron 18 antibióticos con sobras. Los resultados obtenidos demostraron que solo un usuario desconocía el motivo de la receta (Figura 1). Entre los usuarios atendidos en la consulta farmacéutica, cinco ya habían tenido alguna duda sobre una receta en algún momento, cinco supieron explicar de manera resumida qué es la RAM y seis indicaron tener conocimientos sobre los antibióticos. Tres utilizaron antibióticos sin receta en el último año, 14 informaron no tener antibióticos en casa y 13 personas dijeron no saber dónde desechar los medicamentos que sobran de los tratamientos o que están vencidos.

**Figura 1:** Distribución porcentual de las respuestas de los usuarios, referidas al conocimiento sobre antibióticos, recopiladas en consultas farmacéuticas durante el período de enero a abril de 2024 en la UBS Parque Araribá, São Paulo/SP. N = 17.

Fuente: Elaborado por la autora.

Leyenda: Eje x, referente a las preguntas: 1. ¿Sabe por qué se le recetó el medicamento?; 2. ¿Alguna vez ha tenido dudas sobre cómo administrar un medicamento?; 3. ¿Sabe qué es la resistencia a los antimicrobianos?; 4. ¿Sabe qué es un antibiótico?; 5. ¿Ha usado antibióticos sin receta este año?; 6. ¿Suele tener antibióticos en casa?; 7. ¿Sabe dónde desechar los restos o los vencidos?

El formulario farmacoterapéutico utilizado para las consultas constaba de los ejes 1 y 2, correlacionados con los momentos de las consultas.

### Eje 1: Conocimiento Sobre los Antibióticos

Momento de la primera consulta, en el que se evalúa el conocimiento previo del usuario mediante preguntas relacionadas con los antibióticos, la RAM y la eliminación (Figura 1).

### Eje 2: Perfil de los Restos de Medicamento y Devolución

Son los datos relacionados con la segunda consulta (revisión). De los usuarios que recibieron atención en la primera consulta farmacéutica, solo cinco (29,4 %) regresaron para devolver los restos de medicamento, lo que se tradujo en un cumplimiento tras la intervención farmacéutica en cuanto a la devolución correcta de los restos. De estos cinco, solo una persona informó no haber completado el tratamiento de acuerdo con la receta y las indicaciones, aunque la cantidad devuelta coincidía con lo acordado inicialmente.

## DISCUSIÓN

Este estudio muestra que hubo sobras en más del 80 % de las recetas de antibióticos en la UBS Parque Araribá debido a su presentación (envasado) durante el período estudiado. Estas sobras son relativamente grandes y pueden contribuir a la diseminación de la RAM en la comunidad. Sin embargo, en Brasil no existen políticas que regulen de manera efectiva esta dispensación basada en el URM, donde, según la OMS, el usuario debe recibir el medicamento adecuado en la dosis correcta, ajustado a sus necesidades, garantizando el tratamiento completo a un costo razonable.

Los excedentes analizados desde el punto de vista económico indicaron una pérdida financiera de R$ 2.349,54 en el período, con una proyección de más de R$ 7.000,00 para 2024 solo en la UBS Parque Araribá. Estas cifras no llaman la atención por ser un monto exorbitante, pero cuando se aplican a gran escala se pueden estimar pérdidas significativas. Un estudio similar en el municipio de Guarulhos, en 2020, obtuvo una estimación de pérdida de aproximadamente R$ 2.530,26 en una UBS ese año, lo que proyectó para este municipio un desperdicio por sobras estimado en R$ 888.272,04 para el año^(2)^. El municipio de São Paulo cuenta con cerca de 500 servicios de salud que realizan dispensación con posibilidad de generar sobras (UBS y AMA). Al compararlo con el estudio de Guarulhos, podemos suponer que São Paulo podría alcanzar un valor de pérdida financiera aún más relevante, y que estos recursos podrían destinarse a la compra de más medicamentos o a otras inversiones.

Ante un panorama de escasez, resulta cada vez menos aceptable que recursos esenciales, como los antibióticos, se desperdicien de manera irreflexiva por la inacción de las autoridades sanitarias. Sobre todo, el control del consumo de antibióticos es un imperativo para combatir la RAM.

Sin embargo, el perjuicio derivado de los restos de antibióticos va más allá del aspecto meramente financiero, cuando se tiene en cuenta que estos están en manos de una población relativamente desinformada. Nuestros hallazgos demostraron que una parte importante de los entrevistados no supo responder qué era un antibiótico o la RAM, y tampoco sabían dónde desechar los medicamentos que sobraban de tratamientos o que habían vencido. A pesar de que se les indicó que devolvieran los restos, esta acción solo la llevó a cabo una pequeña fracción de las personas que recibieron la orientación. Como consecuencia de los restos de medicamentos en poder de la población, se puede anticipar el posible uso indebido de estos restos por parte del propio usuario o de terceros (vecinos, familiares, niños), considerando que no existe un amplio conocimiento de los riesgos asociados y hay una baja concientización sobre el uso adecuado de los antibióticos^(5)^. En este sentido, se destaca la necesidad de un esfuerzo multidisciplinario para abordar a los pacientes en la atención primaria, reconociendo el papel de otros profesionales además del prescriptor tradicional, que es el médico. Para ello, los equipos deben estar preparados para llevar a cabo estas acciones educativas, teniendo en cuenta el perfil y las necesidades de los pacientes atendidos^(9)^.

Otro factor importante que merece ser destacado es la eliminación inadecuada de los antibióticos. Esto representa un gran riesgo para la salud pública, contribuye a la RAM a través de la contaminación del medio ambiente (suelo, agua y animales) y puede extenderse a toda una comunidad. Las condiciones precarias de saneamiento básico y la vulnerabilidad social, sumadas a la desinformación, contribuyen a la contaminación ambiental por antibióticos^(5)^. Un estudio realizado en Minas Gerais detectó la presencia de estos fármacos en todas las muestras de agua, incluso en áreas con escasa intervención humana, lo que indica que su diseminación va más allá de los centros urbanos^(10)^. De este modo, se destaca la importancia de educar a la población no solo sobre el uso, sino también sobre la eliminación adecuada^(11)^. Otros autores también afirman que el incumplimiento de las normas de envasado de los antibióticos genera una gran cantidad de residuos en la comunidad, lo que contribuye a que la RAM se convierta en un problema de salud, social y económico^(12,13)^.

Como respuesta a esta situación de residuos, la dosificación individualizada y la dispensación precisa parecen ser la solución más adecuada, destacándose como una herramienta significativa. La dispensación individualizada permite que el usuario reciba solo la cantidad exacta necesaria para el tratamiento. Un experimento realizado en farmacias comunitarias de Francia (2014-2015) sustituyó la dispensación de 14 antibióticos en envases originales por la unitarización, lo que redujo la cantidad dispensada, minimizó el desperdicio y favoreció la adherencia al tratamiento^(13,14)^. Sin embargo, la dispensación por unidades de medicamentos en Brasil solo puede realizarse en el lugar de dispensación, en condiciones controladas, de acuerdo con la RDC N.º 80, del 11 de mayo de 2006, lo que exige inversiones y una adaptación significativa de los servicios^(15)^. Otra forma de promover la dispensación unitaria de antibióticos sería la presentación de estos medicamentos en blisters fraccionables; sin embargo, en el país hay pocas marcas y tipos de antibióticos en el mercado.

Sin embargo, en Brasil no existen medidas para regular la producción de presentaciones de medicamentos en envases que sean compatibles con los intereses de la salud pública, ni políticas que regulen la adecuación del mercado farmacéutico y estimulen a los servicios de salud a adquirir antibióticos en dosis individuales o en envases fraccionables, lo que garantizaría la dispensación individualizada. Ese sería el modelo ideal de adquisición de antibióticos en el sistema de salud brasileño, asegurando la dispensación exacta para el tratamiento indicado por el médico.

Mientras no se implemente esta solución más amplia y sensata, una alternativa posible sería establecer condiciones sanitarias para la dispensación unitaria *in loco* en cada farmacia de las Unidades Básicas de Salud (UBS) (o en un centro central o regional), aunque ello genere costos operativos y procesos de trabajo adicionales. Sin embargo, dichas inversiones podrían verse compensadas por la sostenibilidad de una dispensación más precisa.

El farmacéutico desempeña un papel importante como profesional clínico que interviene en la mejora de la adherencia y el manejo de estos medicamentos^(12)^. Las intervenciones farmacéuticas tienen como objetivo garantizar la atención terapéutica integral al usuario. Sin embargo, se enfrentan a obstáculos culturales desafiantes, como la falta de reconocimiento de la importancia del uso correcto de los medicamentos y de la atención farmacéutica, parte importante de su cuidado y tratamiento^(16)^. Además, es fundamental que los profesionales estén calificados

y sean plenamente conscientes de la importancia estratégica de sus funciones. En consecuencia, un autor del Reino Unido señala la necesidad de preparar a los profesionales farmacéuticos para abordar la RAM mediante el uso consciente de los antimicrobianos, incluyendo programas de pregrado que contengan en el plan de estudios materias enfocadas en la administración de antimicrobianos como una competencia obligatoria en las farmacias-escuela de ese país, alineando la educación con la práctica, lo cual podría aplicarse perfectamente al contexto brasileño ^(17)^. Invariablemente, destacamos que la consulta farmacéutica puede sumar esfuerzos a la consulta del enfermero y de otros profesionales de la salud en lo que respecta a la orientación a los usuarios y familiares, potenciando el trabajo en equipo.

Por lo tanto, se necesitan medidas de concientización, educación e incentivo a las políticas públicas para que se preserve la eficacia de los antibióticos para las generaciones futuras^(18)^.

Mientras la dispensación con sobras continúe en los servicios de salud, persistirán el desperdicio financiero y el aumento potencial de la RAM. Es evidente que la atención farmacéutica, integrada al equipo multidisciplinario, puede reducir el desperdicio y promover el uso racional de los medicamentos (URM). Ante este escenario, sin la posibilidad de una dispensación individualizada, se deben tomar otras medidas para intentar minimizar el uso irracional y el desecho indebido de los antibióticos. De esta manera, se propone desarrollar intervenciones eficaces para sensibilizar a los profesionales de la salud y a la población sobre la RAM y mitigar sus factores de riesgo.

Teniendo en cuenta los hallazgos de este estudio, se propuso el desarrollo de material técnico de apoyo orientado a la estandarización del procedimiento de dispensación unitaria y a la implementación de un protocolo para la dispensación individualizada de antibióticos en el ámbito de la APS. Esta iniciativa tiene como objetivo fortalecer aún más el papel del farmacéutico en la atención de la salud, promoviendo una mayor adherencia al tratamiento, minimizando los riesgos de una eliminación inadecuada para el medio ambiente y reduciendo la posibilidad de uso indebido y desperdicio de recursos financieros, mediante la dispensación de antibióticos en estricta conformidad con la posología recetada. Y, en consecuencia, contribuir a mitigar los impactos de los excedentes de antibióticos sobre la RAM en la comunidad. Este material está disponible en una página web^(19)^.

## CONCLUSIÓN

Este estudio demostró la existencia de sobras en las dispensaciones de antibióticos en las unidades de salud. Los resultados de la consulta farmacéutica demostraron que existe un conocimiento insuficiente sobre el uso y la eliminación de los antibióticos. La unitarización de los medicamentos en la APS, combinada con acciones educativas, puede tener un gran potencial para reducir el desperdicio y contribuir a mitigar la RAM.

## Data Availability

Todo el conjunto de datos que respalda los resultados de este estudio se publicó en el propio artículo.
